# Associations of Flavonoid Intakes with Mortality among Populations with Hypertension: A Prospective Cohort Study

**DOI:** 10.3390/nu16101534

**Published:** 2024-05-20

**Authors:** Kang Wang, Taotao Lu, Rukai Yang, Shenghua Zhou

**Affiliations:** 1 Department of Cardiology, Second Xiangya Hospital of Central South University, Changsha 410011, China; wangkang900516@163.com (K.W.); 218202046@csu.edu.cn (R.Y.); 2 Department of Emergency, The First Affiliated Hospital of University of South China, Hengyang 421001, China; m13187308480@163.com

**Keywords:** flavonoid intake, all-cause mortality, CVD-related mortality, cancer-related mortality, hypertension

## Abstract

Background: The effect of flavonoid consumption on all-cause and special-cause mortality remains unclear among populations with hypertension. Methods: A total of 6110 people with hypertension from three NHANES survey cycles (2007–2008, 2009–2010, and 2017–2018) were enrolled in this study. Cox proportional hazard models were conducted to estimate the association between the intake of total flavonoids and flavonoid subclasses and all-cause, cancer-related, and cardiovascular disease (CVD)-related mortality. Nonlinear relationships were identified using restricted cubic splines (RCS). Results: During 43,977 person-years of follow-up, 1155 participants died from any cause, 282 participants died from CVD, and 265 participants died from cancer. After adjusting for relevant confounders, including demographic, lifestyle, and dietary intake, a higher intake of total flavonoids was significantly associated with lower all-cause mortality but not CVD-related and cancer-related mortality among the population with hypertension. Compared with extreme quartiles, the hazard ratio (HR) and 95% confidence interval (CI) were 0.74 (0.56–0.97) for all-cause mortality, 0.77 (0.40–1.46) for CVD-related mortality, and 0.62 (0.35–1.08) for cancer-related mortality. In terms of all-cause mortality, this inverse association was optimized at total flavonoid consumption of approximately 375 mg/day. In addition, the negative association between total flavonoid consumption and all-cause mortality was more pronounced in non-obese (BMI < 30 kg/m^2^) compared to obese (BMI ≥ 30 kg/m^2^) populations. Higher intakes of anthocyanidin, flavan-3-ol, flavonol, and isoflavone were significantly associated with lower all-cause mortality (HR (95%CI): 0.70 (0.55–0.89); 0.76 (0.59–0.96); 0.66 (0.46–0.94); 0.79 (0.67–0.93), respectively). Higher intakes of anthocyanidin, flavan-3-ol, and flavonol were significantly associated with lower cancer-related mortality (HR (95%CI): 0.55 (0.32–0.93); 0.51 (0.31–0.82); 0.52 (0.28–0.96), respectively). Conclusion: This study suggests that a heightened consumption of total flavonoids and some flavonoid subclasses was linked to lower mortality, which supports the proposal of increasing flavonoid intake as part of healthy diets in patients with hypertension.

## 1. Introduction

Hypertension (HTN) is linked to a higher all-cause, cancer- and cardiovascular disease (CVD)-related mortality [1,2]. According to statistics, 1.38 billion people suffered from HTN in 2010, accounting for 31.1% of the world’s adult people [3]. As per the 2017 American College of Cardiology/American Heart Association redefined criteria for HTN, which is systolic blood pressure (SBP) ≥130 mmHg and/or diastolic blood pressure (DBP) ≥80 mmHg, the prevalence of HTN in the U.S. adult population has increased to an amazing 45.4% [4]; however, this new criterion has not yet been agreed upon by other U.S. medical professional societies. With an aging population and increased lifestyle risk factors, such as unhealthy diets, the number of people with HTN continues to rise globally and is projected at 1.56 billion in 2025 [5]. Consistent with the trend of HTN prevalence, the mortality from diseases associated with elevated blood pressure also increased substantially from 1990 to 2015 [6]. Despite effective lifestyle changes and medications, HTN remains the leading cause of mortality from noncommunicable diseases worldwide [7,8,9]. HTN poses a serious global health and financial burden; therefore, simple and effective antihypertensive interventions (e.g., dietary modifications) in managing and reducing the prevalence of HTN should be a priority for global public health systems.

Various healthy diets have been recommended in the HTN management guidelines as an important component of nonpharmacological interventions in recent years [10]. Flavonoids are naturally occurring polyphenolic compounds that include six subclasses, mainly flavanones, flavones, flavan-3-ols, flavonols, isoflavones, and anthocyanins [11]. Owing to their structural diversity, these subclasses exert different effects, such as antioxidant, anti-inflammatory, immunomodulatory, vasodilation, and antithrombotic [11,12,13]. Growing evidence has affirmed that flavonoids, as well as flavonoid-rich foods (e.g., fruit, vegetables, and tea), were linked not only to a reduced risk of HTN but also to lower all-cause, cancer-related, and CVD-related mortality [14,15,16,17,18,19,20]. In addition, flavonoids might offer greater health benefits for people with harmful lifestyles (e.g., smoking and alcohol consumption) [21]. However, the benefits of increased dietary flavonoid intake in patients with HTN remain unclear. Further studies are required to determine the interrelationships between flavonoid consumption and all-cause, cancer-related, and CVD-related mortality and to establish the appropriate dose required to achieve optimal benefit in patients with HTN.

The primary aim of this study was to investigate the association of the intake of total flavonoids and flavonoid subclasses with all-cause, cancer-related, and CVD-related mortality in 6110 adults with HTN from the National Health and Nutrition Examination Survey (NHANES). The results showed that the intake of total flavonoids was significantly linked to reduced all-cause mortality but not to CVD-related and cancer-related mortality among the population with HTN. Among the flavonoid subclasses, there were significant associations between anthocyanins, flavan-3-ols, and flavonols and all-cause and cancer-related mortality, as well as between isoflavones and all-cause mortality.

## 2. Materials and Methods

### 2.1. Study Population

**Figure 1:**
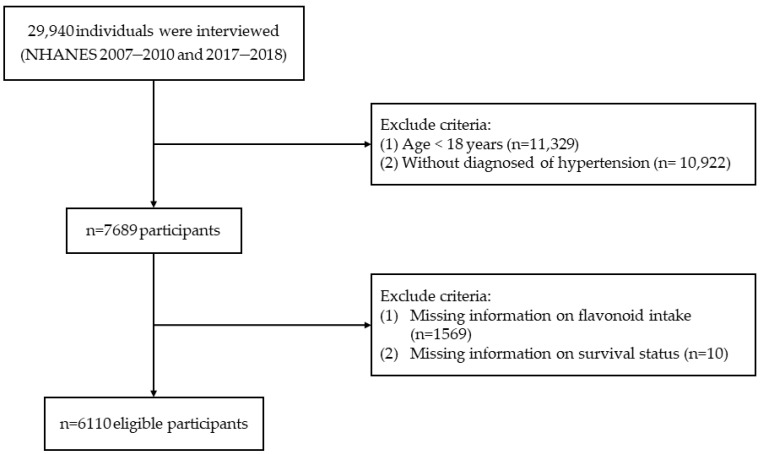
Flow chart of participant selection.

We identified a total of 29,940 individuals from 3 survey cycles (2007–2008, 2009–2010, and 2017–2018) of NHANES data. Exclusions were made for individuals under 18 years old (*n* = 11,329), those without HTN (*n* = 10,922), those lacking flavonoid dietary data (*n* = 1569), and without information from survival status (*n* = 10). Finally, the study enrolled 6110 participants (Figure 1). The diagnosis of HTN included fulfilling one of the following conditions: (i) a history of HTN; (ii) being on an antihypertensive regimen; (iii) with an average SBP ≥ 140 mmHg or average DBP ≥ 90 mmHg [22]. The National Center for Health Statistics (NCHS) Research Ethics Review Board approved the NHANES protocol [23]. All participants signed informed consent.

### 2.2. Assessment of Flavonoid Intake

Flavonoid intake values of foods and beverages were obtained from the United States Department of Agriculture (USDA) Food and Nutrient Database for Dietary Studies (FNDDS) [24] and corresponding dietary data from the NHANES [25]. Flavonoid content (mg/100 g) of every beverage/food was established by the USDA Nutrition Data Laboratory [26]. The intake of flavonoids was collected through two 24 h dietary recall interviews. We used the average of the sum of day 1 and day 2 dietary flavonoid intakes, including the 6 major flavonoid subclasses: (1) Total anthocyanidins (petunidin, peonidin, malvidin, delphinidin, pelargonidin, and cyanidin); (2) Total isoflavones (glycitein, genistein, and daidzein); (3) Total flavonols (quercetin, kaempferol, isorhamnetin, and myricetin); (4)Total flavones (apigenin and luteolin); (5) Total flavanones (naringenin, eriodictyol, and hesperetin); (6) Total flavan-3-ols [theaflavin-3′-gallate, theaflavin-3-gallate, (-)-epigallocatechin, (+)-gallocatechin, theaflavin, theaflavin-3-3′-digallate, (-)-epicatechin, (-)-epigallocatechin 3-gallate, (+)-catechin, (-)-epicatechin 3-gallate, and thearubigins] [27].

### 2.3. Mortality Confirmation

The deadline for follow-up was 12.31.2019 [28]. The follow-up duration was ascertained by the time in months from the household interview to the death or loss of follow-up of the participant. Survival status was defined through the National Death Index. The International Classification of Diseases, tenth revision (ICD-10) defined the cause of death. All-cause deaths were defined as deaths from any cause. Cancer-related deaths were defined as C00-C97. CVD-related deaths were defined as I00-I09, I11, I13, I20-I51, and I60-I69.

### 2.4. Covariates Assessment

Most of the variables were collected through the interview questionnaire: age, race, activity, alcohol consumption, marital status, education, smoking status, and dietary intake (including fruit, vegetables, whole grains, and red meat). Marital status was categorized as with partner and without partner. Educational status was divided into <9 years, 9–12 years, and >12 years. Smoking status was divided into three groups: “never” marked by ≤100 cigarettes in a lifetime; “former” defined as >100 cigarettes in a lifetime and none smoked at all now; and “now” defined as >100 cigarettes in a lifetime and smoked some days or every day. Activity levels were categorized as inactive, moderate, and vigorous. Alcohol consumption, total daily energy intake, and total fruit, vegetables, whole grains, and red meat intake were averaged as the sum of days 1 and 2. The formula, weight (kg)/height (m^2^), was utilized in determining the body mass index (BMI).

Diabetes was diagnosed in each of the following conditions: (i) random glucose content ≥11.1 mmol/L; (ii) HbA1c concentration ≥6.5%; (iii) fasting glucose level ≥7.0 mmol/L; (iv) oral glucose tolerance test ≥11.1 mmol/L; or (v) the use of antidiabetic drugs [27]. Each of the following conditions was diagnosed as hyperlipemia: (i) total cholesterol ≥5.2 mmol/L; (ii) triglyceride ≥1.7 mmol/L; (iii) low-density lipoprotein ≥3.4 mmol/L; (iv) high-density lipoprotein ≤1.3 mmol/L for women and ≤1.0 mmol/L for men; (v) or use of cholesterol-lowering drugs [29].

### 2.5. Statistical Analysis

All analyses included sample weights to consider the complex sampling design of the NHANES [30]. Pearson’s correlation coefficient analyzed the correlation between flavonoid subclasses.

The Cox proportional hazard models estimated the association between the intake of total flavonoids and flavonoid subclasses and all-cause, cancer-related, and CVD-related mortality. To account for potential confounding, we adjusted for age (continuous, years) and sex (female and male) in model 1. In model 2, we further adjusted for race (White, Black, Hispanic, Mexican American, and others), marital status (without partner and with partner), education status (<9 years, 9–12 years, and >12 years), activity (vigorous, moderate, and inactive), smoking status (former, current, and never), alcohol intake (continuous, g/d), BMI (continuous, kg/m^2^) and daily energy consumption (continuous, kcal/d). In model 3, we further adjusted for total fruit intake (continuous, cup/d), total vegetable intake (continuous, cup/d), whole grains intake (continuous, oz/d), and red meat intake (continuous, oz/d). Potential nonlinear relationships were analyzed using restricted cubic splines (RCS) regression with three knots at the 10th, 50th, and 90th centiles.

Furthermore, we conducted stratified analysis by several key risk factors, including age (<65, ≥65 years), sex (male, female), BMI (<30, ≥30 kg/m^2^), marital status (without and with partner), activity (inactive and moderate/vigorous), alcohol intake (<10, ≥10 g/day), smoking status (never and former/current smoker), and education status (≤12 and >12 years) by incorporating an interaction term into the completely adjusted model. The interaction was tested in these stratified variables by the likelihood-ratio test.

Sensitivity analysis was executed to test the robustness of our research findings. First, we additionally adjusted for chronic diseases at the baseline, including diabetes and hyperlipemia. Second, we excluded participants with extreme energy intake at the baseline (<500 or >4000 kcal/day). Finally, we reanalyzed the data by excluding individuals with ≤1 year of follow-up.

All statistical tests were performed with R software (v4.3.1), and *p* < 0.05 was deemed statistically significant.

## 3. Results

### 3.1. Subsection

#### 3.1.1. Baseline Characteristics

The 6110 NHANES participants represented 83.2 million noninstitutionalized residents of the U.S. in 2007–2010 and 2017–2018. The average age of the participants at the baseline was 57.7 (SD 0.4) years, of which 3117 (50.9%) were female and 2870 (69.2%) were White. During 43,977 person-years of follow-up, 1155 participants died from any cause, 282 participants died from CVD, and 265 participants died from cancer. The baseline characteristics of the study population are visualized in Table 1, categorized into quartiles as per their total flavonoid consumption. Compared with the participants with lower total flavonoid consumption, those with higher total flavonoid consumption tended to be White, better educated, and partnered. Supplementary Table S1 manifests the distributional characteristics of participants’ total and flavonoid subclass intake. Correlations between flavonoid subclasses were assessed and are shown in Supplementary Table S2. Except for the intake of isoflavone and the intake of other flavonoid subclasses, and the intake of flavanone and the intake of flavan-3-ols, the flavonoid subclasses were significantly associated with each other (*p* < 0.05).

#### 3.1.2. Total Flavonoid and Flavonoid Subclasses and All-Cause Mortality

After adjusting for multiple covariates (Model 3), participants in the highest quartile of total flavonoid consumption had a lower risk of all-cause mortality in comparison to those in the lowest intake quartile. The HR and 95%CI were 0.74 (0.56–0.97) (Table 2). In addition, we observed that increased consumption of anthocyanidins, flavan-3-ols, isoflavones, and flavonols was linked to lower all-cause mortality in all subclasses. The HRs and 95%CIs of comparing the extreme groups were 0.70 (0.55–0.89) for total anthocyanidins, 0.76 (0.59–0.96) for total flavan-3-ols, and 0.66 (0.46–0.94) for total flavonols (Table 3). Since 40.1% of the participants did not consume isoflavone, the cohort was categorized into two groups according to median isoflavone intake. We also observed a similar association (0.79 (0.67–0.93)) (Table 4).

**Figure 2:**
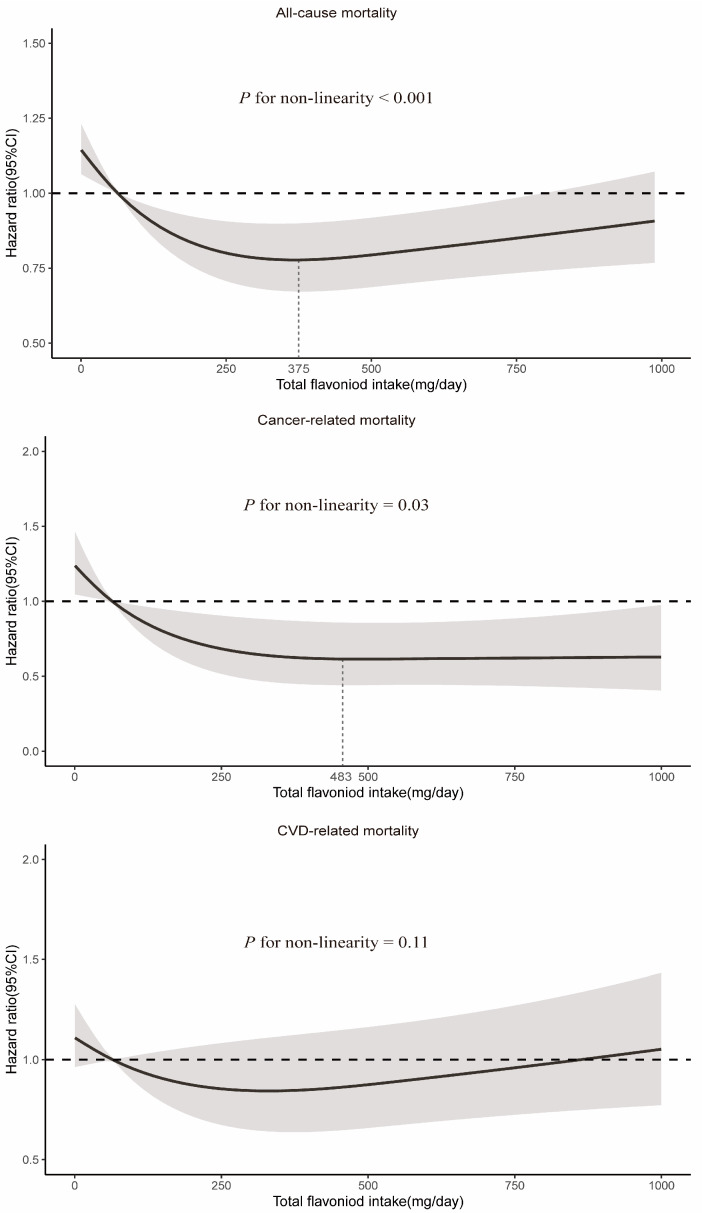
Dose–response relationships between total flavonoid intake and all-cause and cause-specific mortality. Median total flavonoid intake is reference standard. Hazard ratios are based on Cox proportional hazards models adjusted for age (continuous, years), sex (female and male), race (White, Black, Hispanic, Mexican American, and others), marital status (without partner and with partner), education status (<9 years, 9–12 years, and >12 years), activity (vigorous, moderate, and inactive), smoking status (never, former, and current), alcohol intake (continuous, g/d), BMI (continuous, kg/m^2^), daily energy intake (continuous, kcal/d), total fruit intake (continuous, cup/d), total vegetable intake (continuous, cup/d), whole grains intake (continuous, oz/d), and red meat intake (continuous, oz/d). Solid lines indicate HR and shadows indicate 95%CI.

**Figure 3:**
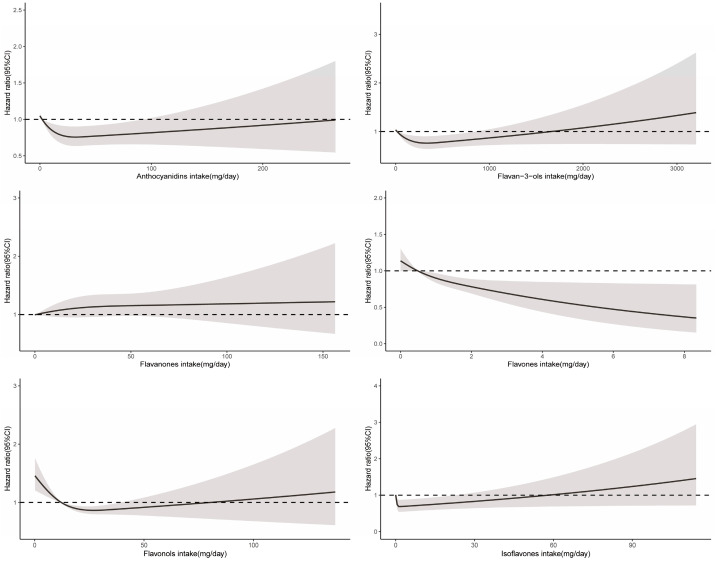
Dose–response relationships between flavonoid subclass intake and all-cause mortality. Median flavonoid subclasses intake is reference standard. Hazard ratios are based on Cox proportional hazards models adjusted for age, sex, race, marital status, education status, activity, smoking status, alcohol intake, BMI, daily energy intake, total fruit intake, total vegetable intake, whole grains intake, and red meat intake. Solid lines indicate HR and shadows indicate 95%CI.

RCS demonstrated a significant nonlinear relationship between total flavonoid consumption and all-cause mortality (*P*_non-linearity_ < 0.001), with evidence for optimal effect at a consumption level of approximately 375 mg/day (Figure 2). Similarly, significant nonlinear associations were observed for some flavonoid subclasses (including flavan-3-ols, anthocyanidins, flavonols, and isoflavones), with the optimal intake doses occurring at different levels of intake (322 mg/d, 33 mg/d, 26 mg/d, and 2 mg/d, respectively). In contrast, the intake of flavanones and flavones demonstrated a linear association with all-cause mortality (Figure 3).

In addition, we also analyzed the association between 29 flavonoids and all-cause mortality, respectively. The intake of genistein, apigenin, glycitein, myricetin, peonidin, epigallocatechin-3-gallate, epicatechin-3-gallate, kaempferol, epigallocatechin, quercetin, and gallocatechin were linked to decreased all-cause mortality (Supplementary Table S3).

#### 3.1.3. Total Flavonoid and Flavonoid Subclasses and Cause-Specific Mortality

We analyzed the association between total flavonoids and flavonoid subclasses with cancer-related and CVD-related mortality, respectively. For cancer-related mortality, there was no significant link from the total flavonoid intake in the fully adjusted model (Model 3) (0.62 (0.35–1.08)) (Table 2). However, anthocyanidins, flavan-3-ols, and flavonols among the flavonoid subclasses were significantly associated with lower cancer-related mortality. The HRs and 95%CIs of comparing the extreme groups were 0.55 (0.32–0.93) for total anthocyanidins, 0.51 (0.31–0.82) for total flavan-3-ols, and 0.52 (0.28–0.96) for total flavonols (Table 3).

**Figure 4:**
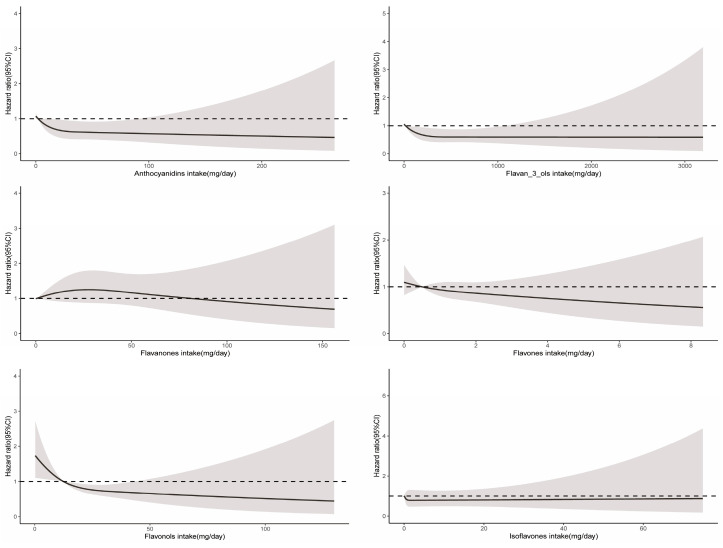
Dose–response relationships between flavonoid subclass intake and cancer-related mortality. Median flavonoid subclasses intake is reference standard. Hazard ratios are based on Cox proportional hazards models adjusted for age, sex, race, marital status, education status, activity, smoking status, alcohol intake, BMI, daily energy intake, total fruit intake, total vegetable intake, whole grains intake, and red meat intake. Solid lines indicate HR and shadows indicate 95%CI.

A significant nonlinear relationship was observed between the consumption of total flavonoids and cancer-related mortality (*P*_non-linearity_ = 0.03), and the optimal intake of total flavonoids was approximately 483 mg/day (Figure 2). The intake of all six flavonoid subclasses showed a linear relationship with cancer mortality (*P*_non-linearity_ > 0.05). As depicted in Figure 4, the intake of more flavonoids was associated with lower cancer-related mortality. In addition, for each flavonoid, the intake of delphinidin, myricetin, kaempferol, malvidin, peonidin, epigallocatechin, theaflavin, thearubigins, theaflavin-3′-gallate, quercetin, theaflavin-3-gallate, gallocatechin, theaflavin-3-3′-digallate, and subtotal catechins were all associated with lower cancer-related mortality (Supplementary Table S4).

**Figure 5:**
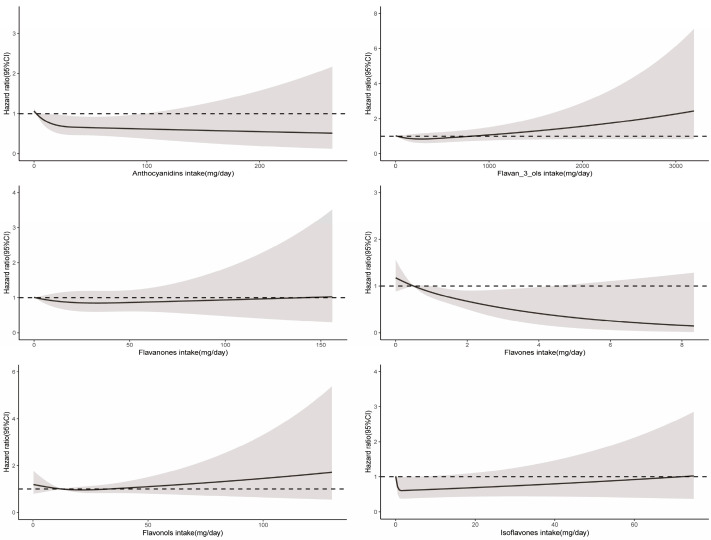
Dose–response relationships between flavonoid subclass intake and CVD-related mortality. Median flavonoid subclasses intake is reference standard. Hazard ratios are based on Cox proportional hazards models adjusted for age, sex, race, marital status, education status, activity, smoking status, alcohol intake, BMI, daily energy intake, total fruit intake, total vegetable intake, whole grains intake, and red meat intake. Solid lines indicate HR and shadows indicate 95%CI.

For CVD-related mortality, no significant association from the total flavonoid intake was found (Table 2). When adjusted for age and sex (Model 1), compared with the lowest intake group, the group with the greatest consumption of anthocyanidins, flavanones, flavones, and isoflavones was linked to lower CVD-related mortality, but this association was significantly attenuated after further adjustment for lifestyle and dietary factors (Table 3 and Table 4). Isoflavones showed significant nonlinear relationships for CVD-related mortality (*P*_non-linearity_ = 0.02) (Figure 5). Glycitein intake only was linked to lower CVD-related mortality among 29 flavonoids (Supplementary Table S5).

#### 3.1.4. Stratified Analyses

In stratified analyses, we examined the interrelationships between total flavonoids consumption and all-cause and cause-specific mortality across subgroups defined by age (<65, ≥65 years), sex (male, female), BMI (<30, ≥30 kg/m^2^), marital status (without and with partner), activity (inactive and moderate/vigorous), alcohol intake (<10, ≥10 g/day), smoking status (never and former/current smoker), and education status (≤12 and >12 years).

**Figure 6:**
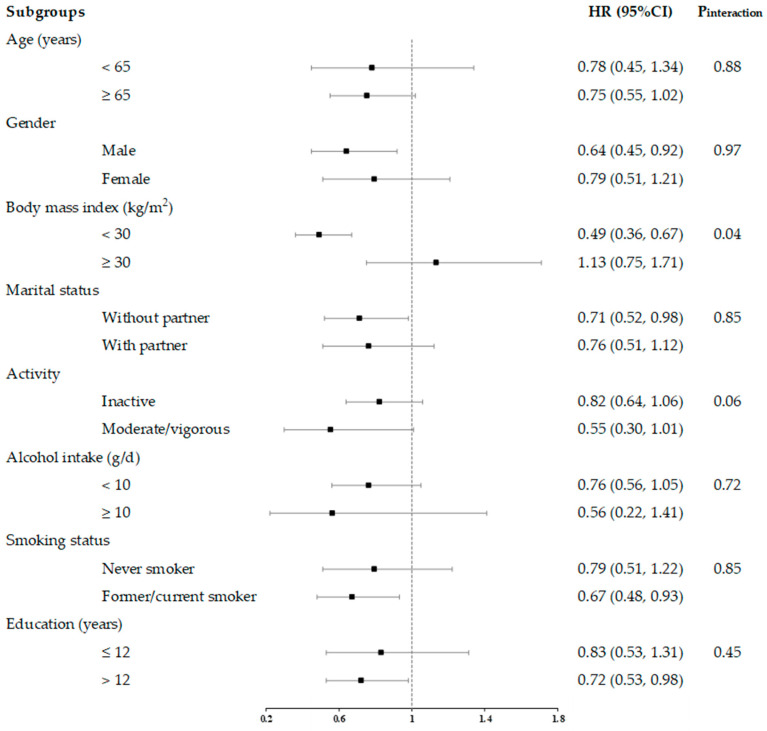
HRs and 95% CIs for consumption of total flavonoids and all-cause mortality, stratified by several key risk factors. All models were multivariable adjusted for age, sex, race, marital status, education status, activity, smoking status, alcohol intake, BMI, daily energy intake, total fruit intake, total vegetable intake, whole grains intake, and red meat intake. In each stratified analysis, the stratification variable was excluded in the adjustments. The HR and 95%CI of each subgroup in the figure from the group with the highest intake of total flavonoids is shown. Likelihood ratio tests were used for assessment of interaction, and two-sided *p* values (unadjusted for multiple comparisons) are reported.

In general, the association between lower all-cause mortality and higher total flavonoid consumption was more pronounced in non-obese populations (BMI < 30 kg/m^2^) than in obese populations (BMI ≥ 30 kg/m^2^) (Figure 6). For cancer-related mortality, we found stronger associations in the active populations than in the inactive populations (Supplementary Figure S1). For CVD-related mortality, we observed consistent associations between these subgroups (Supplementary Figure S2).

#### 3.1.5. Sensitivity Analyses

The results of the sensitivity analyses demonstrated the robustness of the findings of this study. First, further adjustments for other chronic diseases (including diabetes and hyperlipidemia) did not substantively alter the results (0.77 (0.58–0.99)) (Supplementary Table S6). Second, no differences were observed in the results after the participants with extreme energy intake (<500 or >4000 kcal/day) were excluded (0.76 (0.58–0.99)) (Supplementary Table S7). Finally, the results did not substantially alter after the participants with ≤1 year of follow-up were excluded (0.72 (0.54–0.95)) (Supplementary Table S8).

## 4. Discussion

### 4.1. Main Findings

There is considerable potential for improving public health by promoting healthier dietary behaviors and tailoring the dietary regimens for individuals with HTN by understanding the efficacy of specific dietary components. This prospective cohort study provides evidence that a higher intake of total flavonoids has positive interrelationships with lower risk of all-cause mortality and that the optimal intake of them is approximately 375 mg/day. The results additionally revealed that inverse associations between total flavonoid consumption and all-cause mortality are more pronounced in non-obese (BMI < 30 kg/m^2^) versus obese (BMI ≥ 30 kg/m^2^) populations. In addition, the study found lower all-cause mortality was associated with the intake of anthocyanidins, flavan-3-ols, flavonols, and isoflavones. Lower cancer-related mortality was associated with the intake of anthocyanidins, flavan-3-ols, and flavonols.

### 4.2. Comparison with Previous Studies

This study examined the relationship between all-cause and cause-specific mortality and the dietary habits of individuals with HTN. Although direct comparison with other studies cannot be drawn, previous studies on related topics exist, such as the interrelationship between the intake of flavonoids and the risk of HTN and between flavonoid intake and all-cause and cause-specific mortality in the general population. The European Prospective Investigation into Cancer and Nutrition (EPIC) cohort, a prospective study of French women, reported that a higher total flavonoid consumption was linked to a lower incidence of HTN risk in women (HR of comparing two extreme quintiles: 0.91, 95% CI 0.85–0.97) [17]. In addition, in flavonoid subclass analyses, an increased consumption of anthocyanidins and flavonols was linked to a lower risk of HTN [17]. However, another prospective study including three cohorts found different results, with the intake of total flavonoids not significantly associated with the risk of HTN, and only anthocyanidins among the flavonoid subclasses were linked to a lower risk of HTN (HR 0.92, 95%CI 0.86–0.98) [15]. Consistently, a recent study, also from the NHANES, observed similar results. The intake of anthocyanidins but not total flavonoids was significantly linked to a lower risk of HTN (HR 0.81, 95%CI 0.66–0.99) [31]. Overall, anthocyanidin intake is beneficial in reducing the risk of HTN. We further found the benefits of anthocyanidins on mortality among individuals with HTN. A most recent meta-analysis incorporating prospective cohort studies constituting 462,194 participants from 16 cohorts inferred that compared with those in the lowest category, those in the highest total flavonoid intake category showed a 13% reduction in all-cause mortality (relative risk (RR): 0.87, 95%CI = 0.77–0.99) and a 15% decline in CVD-related mortality (0.85, 0.75–0.97), but not cancer-related mortality [19]. However, our study did not find a significant association between total flavonoids and CVD-related mortality. In addition, there are several prospective cohort studies from the same NHANES database. In line with our results, Zhou et al. reported that total flavonoids was not significantly associated with cancer-related mortality. Furthermore, this cohort study also found that the intake of total flavonols was inversely associated with cancer-related mortality (HR: 0.54, 95%CI = 0.30–0.99) [27]. Another study from the NHANES database found that flavonols, anthocyanidins, and isoflavones were all significantly negatively associated with all-cause mortality. The HRs and 95%CIs were 0.87 (0.81–0.94) for flavonols, 0.91 (0.84, 0.99) for anthocyanidins, and 0.81 (0.70, 0.94) for isoflavones, respectively. However, no significant association was found between total flavonoid intake and all-cause mortality and CVD-related mortality [32]. Furthermore, Zong et al. specifically examined the association of flavonols with all-cause and cause-specific mortality and found that the intake of flavonols was strongly associated with lower all-cause (HR: 0.64, 95%CI = 0.54–0.75), CVD-related (0.67, 0.47–0.96), and cancer-related mortality (0.45, 0.28–0.70) [33]. In summary, our findings are partially different from the studies mentioned above, probably due to differences in the study populations. In fact, the population with HTN was more strongly associated with higher mortality [1]. Our study focused specifically on this group with specific diseases and a high risk of mortality.

There is a threshold of dietary intake for optimal effects, and higher intakes do not provide more benefits. Previous studies also support this idea. A meta-analysis encompassing 15 prospective cohort studies demonstrated a statistically significant dose–response nonlinear relationship between total flavonoid consumption and all-cause mortality, with a threshold intake of 200 mg/day [34]. Our findings affirm that the optimal dose of total flavonoid intake associated with all-cause was 375 mg/day. This value was higher than the results of previous studies, perhaps because there is a higher mortality in people with HTN compared to the general population, so higher doses of dietary flavonoids are required to achieve the optimal benefit. Flavonoid-rich foods include fruits and vegetables, tea, wine, and chocolate [35]. A cup of tea, 100 g of blueberries, an orange, an apple, and 100 g of broccoli are sufficient to meet 500 mg flavonoid intake [36]. 

Moreover, we observed that the negative association between the total flavonoid intake and all-cause mortality was more pronounced in the non-obese population, whereas the negative association between the total flavonoid intake and cancer-related mortality was more pronounced in the inactive population. These results indicate that the total flavonoid intake may not be able to counteract the harmful effects of unhealthy lifestyles.

### 4.3. Public Health and Clinical Implications

The implications of our study are noteworthy not only to the clinical practice but also to the public health. The prevalence of HTN increased considerably from 1990 to 2015, with the increasing global population and aging individuals, and it remains the major cause of death from noncommunicable diseases worldwide [6]. About 8.4% of deaths due to noncommunicable diseases can be attributed to a low intake of fruits or vegetables [21]. The successful treatment of HTN should combine measures at the pharmacologic and nonpharmacologic levels, while nonpharmacologic therapy should be used throughout life [4,37,38]. Although numerous previous studies have clarified the risk factors for HTN, few studies have focused on the benefits of nonpharmacologic interventions (e.g., dietary modifications) for premature death in patients with HTN. In other words, previous investigations explored the link between flavonoids and the risk of mortality were conducted among the general population without specifically focusing on individuals with HTN [21,27,32,36,39]. Thus, it remains unclear whether a high consumption of total and flavonoid subclasses is linked to lower all-cause, cancer-related, and CVD-related mortality in populations with HTN. This is the first study to delve into the interrelationship between total flavonoids and flavonoid subclasses and all-cause, cancer-related, and CVD-related mortality in the population with HTN, providing additional data to the existing literature in this field. In addition, although public health nutrition guidelines recommend the importance of healthy dietary patterns for the management of HTN, these rarely specify the optimal amount of each food or dietary component required, which may be one of the reasons why the rates of awareness and control of HTN remain unsatisfactory. Furthermore, several surveys have shown that even the countries with the highest control rates—namely, the United States, Canada, and Germany—are below 70% in the 49–79 years age group [9]. This study supports the public health and clinical recommendations that increasing dietary flavonoid intake in patients with HTN is beneficial in further reducing all-cause, cancer-related, and CVD-related mortality and provides the optimal dose of flavonoids.

### 4.4. Mechanisms

The benefits of flavonoids may be related to their multifaceted bioactivities, including antioxidant [40,41], anti-inflammatory [42,43], anti-platelet aggregation [44,45], modulating gut microbiota [46], and vasodilation [13,47]. Although their bioavailability is limited, this shortcoming is compensated by their potent bioactivities and diverse biological effects. Firstly, oxidative stress is strongly associated with HTN and HTN-induced organ damage [48]. Flavonoids are strong antioxidants that prevent the damage of oxidative stress [40,41], with anthocyanidins showing the highest antioxidant activity [49]. However, recent studies have found that flavonoids may also exhibit pro-oxidant effects, depending on their concentration and the environment (e.g., PH) [49,50]. Therefore, the way in which flavonoids exert optimal health benefits still needs to be further explored. Secondly, inflammation can lead to higher mortality [51]. Flavonoids can affect arachidonic acid metabolism by inhibiting the expression of cyclooxygenase 2 and lipoxygenase, ultimately exerting the anti-inflammatory effect [42,43]. Finally, flavonoids can also exert antihypertensive effects by increasing the bioavailability of nitric oxide (NO) and modulating vasoactive factors and vasodilation [13,47], and this process may also involve alterations of metabolites associated with gut microbiota [46], such as 3-hydroxyphenylacetic acid [52].

### 4.5. Strengths and Limitations

The prospective study design and multiple confounding variable adjustments mark the strengths of this study. Designing subgroup analyses and sensitivity analyses improved the robustness of the findings. In addition, we used 2-day intake data for each flavonoid and constructed weights to better reflect the entire U.S. population.

However, several limitations of this study must be considered. First, as this is an observational study, inference of causality is not possible. Second, dietary data collected at the baseline may lead to misclassification because dietary habits are likely to change during long-term follow-up. Third, although we adjusted for relevant confounding variables as much as possible in the model, residual confounders could not be entirely excluded.

## 5. Conclusions

Utilizing a nationally representative prospective cohort study, we observed the link between higher total flavonoid intake and lower all-cause mortality in populations with HTN. From public health and clinical perspectives, the findings of this study further demonstrate the potential to lower mortality in patients with HTN through higher dietary flavonoid intake. However, we still need a series of randomized controlled trials to further elucidate the health benefits of flavonoids.

## Figures and Tables

**Table 1:** Baseline characteristics of the participants by intake of total flavonoids.

	Total Flavonoid Intake (mg/d)				
	Overall	Q1 <23.9	Q2 23.9–63.1	Q3 63.1–212.5	Q4 >212.5
No. of participants	6110	1528	1527	1527	1528
Age (years)	57.7 (0.4)	54.7 (0.7)	57.6 (0.7)	60.2 (0.5)	58.2 (0.6)
BMI (kg/m^2^)	31.3 (0.2)	32.0 (0.3)	31.4 (0.3)	30.9 (0.3)	31.2 (0.3)
Daily energy intake (kcal/d)	2013 (17)	1765 (25)	2027 (27)	2148 (32)	2090 (38)
Alcohol consumption (g/d)	8.9 (0.5)	4.9 (0.7)	11.7 (1.0)	10.4 (1.0)	8.3 (1.0)
Fruit intake (cup/d)	1.0 (0.0)	0.3 (0.0)	0.9 (0.0)	1.5 (0.1)	1.2 (0.1)
Vegetable intake (cup/d)	1.5 (0.0)	1.1 (0.0)	1.6 (0.1)	1.7 (0.1)	1.7 (0.0)
Whole grains intake (oz/d)	0.8 (0.0)	0.6 (0.0)	0.9 (0.0)	1.0 (0.0)	0.9 (0.1)
Red meat intake (oz/d)	1.7 (0.1)	1.5 (0.1)	1.6 (0.1)	1.7 (0.1)	1.8 (0.1)
Sex, %					
Female	3117 (50.9)	756 (49.9)	769 (47.5)	796 (51.4)	796 (54.3)
Male	2993 (49.1)	772 (50.1)	758 (52.5)	731 (48.6)	732 (45.7)
Race, %					
White	2870 (69.2)	681 (66.0)	704 (68.2)	692 (68.0)	793 (73.9)
Black	1571 (14.0)	447 (17.3)	371 (13.8)	385 (14.0)	368 (11.5)
Hispanic	529 (4.7)	138 (4.6)	160 (5.9)	148 (5.9)	83 (2.7)
Mexican American	729 (5.8)	181 (6.9)	213 (6.9)	193 (5.6)	142 (4.1)
Others	411 (6.3)	81 (5.3)	79 (5.3)	109 (6.4)	142 (7.8)
Education, %					
<9 years	711 (6.4)	233 (8.9)	191 (6.8)	179 (6.3)	109 (4.1)
9–12 years	952 (11.3)	295 (16.0)	242 (11.4)	204 (8.9)	211 (9.5)
>12 years	4434 (82.1)	998 (75.1)	1093 (81.9)	1136 (84.8)	1207 (86.3)
Activity, %					
Inactive	3738 (55.3)	963 (55.9)	954 (60.7)	923 (53.1)	898 (52.1)
Moderate	1287 (24.6)	279 (21.6)	299 (20.5)	356 (27.9)	353 (27.8)
Vigorous	1085 (20.0)	286 (22.4)	274 (18.8)	248 (18.9)	277 (20.1)
Marital status, %					
Without partner	2500 (34.9)	678 (38.8)	646 (37.6)	617 (33.3)	559 (31.6)
With partner	3570 (64.5)	837 (61.2)	873 (62.4)	902 (66.7)	958 (68.4)
Smoke, %					
Never	3057 (51.1)	655 (43.5)	761 (51.2)	840 (56.2)	801 (53.7)
Former	1946 (31.3)	476 (31.2)	488 (31.5)	506 (32.9)	476 (30.2)
Current	1079 (17.2)	391 (25.3)	271 (17.3)	173 (10.9)	244 (16.2)
Diabetes, %					
No	4122 (73.0)	974 (70.3)	1019 (71.9)	1069 (75.6)	1060 (74.9)
Yes	1974 (26.7)	549 (29.7)	507 (28.1)	454 (24.4)	465 (25.1)
Hyperlipidemia, %					
No	1160 (18.2)	321 (21.3)	285 (18.6)	284 (18.4)	270 (15.2)
Yes	4949 (81.8)	1207 (78.7)	1242 (81.4)	1242 (81.6)	1258 (84.8)

Data expressed as mean [SD] or n (%) unless otherwise stated. Abbreviations: BMI, body mass index.

**Table 2:** HRs and CIs of all-cause, CVD-related, and cancer-related mortality by quartiles of flavonoid intake.

	Total Flavonoid Intake			
	Q1	Q2	Q3	Q4
Total flavonoids (mg/day)	12.6 (0.2)	40.9 (0.4)	118.0 (1.8)	665.8 (27.5)
Case/Person-years	1528/10,707	1527/11,071	1527/10,706	1528/11,494
All-cause mortality				
No. events (n)	318	286	294	257
Model 1	ref	0.75 (0.63, 0.89)	0.59 (0.49, 0.73)	0.60 (0.47, 0.76)
Model 2	ref	0.86 (0.71, 1.04)	0.75 (0.59, 0.96)	0.75 (0.59, 0.97)
Model 3	ref	0.85 (0.69, 1.04)	0.72 (0.55, 0.96)	0.74 (0.56, 0.97)
CVD-related mortality				
No. events (n)	65	76	79	62
Model 1	ref	0.64 (0.41, 0.99)	0.63 (0.42, 0.94)	0.60 (0.35, 1.02)
Model 2	ref	0.71 (0.45, 1.12)	0.75 (0.48, 1.17)	0.75 (0.42, 1.33)
Model 3	ref	0.73 (0.45, 1.17)	0.78 (0.46, 1.33)	0.77 (0.40, 1.46)
Cancer-related mortality				
No. events (n)	80	64	62	59
Model 1	ref	0.92 (0.55, 1.53)	0.54 (0.33, 0.88)	0.51 (0.31, 0.84)
Model 2	ref	1.07 (0.65, 1.78)	0.68 (0.40, 1.16)	0.60 (0.35, 1.01)
Model 3	ref	1.10 (0.65, 1.86)	0.68 (0.37, 1.24)	0.62 (0.35, 1.08)

Hazard ratios (95% CI) for all-cause, CVD-related, and cancer-related mortality were analyzed using Cox proportional hazard models. Model 1: age (continuous, years) and sex (female and male). Model 2: model 1 + race (White, Black, Hispanic, Mexican American, and others), marital status (without partner and with partner), education status (<9 years, 9–12 years, and >12 years), activity (vigorous, moderate, and inactive), smoking status (never, former, and current), alcohol intake (continuous, g/d), BMI (continuous, kg/m^2^), and daily energy intake (continuous, kcal/d). Model 3: model 2 + total fruit intake (continuous, cup/d), total vegetable intake (continuous, cup/d), whole grains intake (continuous, oz/d), and red meat intake (continuous, oz/d).

**Table 3:** HRs and CIs of all-cause, CVD-related, and cancer-related mortality by quartiles of six flavonoid subclass intakes.

	Total Flavonoid Subclass Intake			
	Q1	Q2	Q3	Q4
Anthocyanidins (mg/day)	≤0.12	0.12–1.99	1.99–10.40	>10.40
Case/Person-years	1528/10,445	1528/11,135	1526/11,425	1528/10,973
All-cause mortality				
No. events (n)	295	309	290	261
Model 1	ref	0.86 (0.69, 1.07)	0.69 (0.54, 0.87)	0.57 (0.46, 0.71)
Model 2	ref	1.01 (0.81, 1.25)	0.84 (0.67, 1.07)	0.73 (0.59, 0.92)
Model 3	ref	1.00 (0.80, 1.25)	0.82 (0.63, 1.06)	0.70 (0.55, 0.89)
CVD-related mortality				
No. events (n)	70	72	70	70
Model 1	ref	0.91 (0.54, 1.52)	0.67 (0.41, 1.10)	0.53 (0.32, 0.90)
Model 2	ref	1.09 (0.67, 1.78)	0.85 (0.50, 1.46)	0.66 (0.38, 1.16)
Model 3	ref	1.10 (0.65, 1.84)	0.84 (0.47, 1.52)	0.65 (0.33, 1.25)
Cancer-related mortality				
No. events (n)	75	76	57	57
Model 1	ref	0.76 (0.48, 1.21)	0.57 (0.36, 0.91)	0.42 (0.26, 0.68)
Model 2	ref	0.89 (0.56, 1.40)	0.75 (0.48, 1.18)	0.56 (0.34, 0.93)
Model 3	ref	0.90 (0.58, 1.41)	0.74 (0.46, 1.18)	0.55 (0.32, 0.93)
Flavan-3-ols (mg/day)	≤4.90	4.90–15.18	15.18–153.29	>153.29
Case/Person-years	1528/10,645	1527/11,038	1527/10,750	1528/11,545
All-cause mortality				
No. events (n)	315	309	268	263
Model 1	ref	0.80 (0.67, 0.96)	0.66 (0.53, 0.81)	0.63 (0.50, 0.79)
Model 2	ref	0.93 (0.78, 1.11)	0.81 (0.68, 0.96)	0.77 (0.61, 0.98)
Model 3	ref	0.91 (0.76, 1.08)	0.77 (0.63, 0.93)	0.76 (0.59, 0.96)
CVD-related mortality				
No. events (n)	66	75	68	73
Model 1	ref	1.09 (0.73, 1.64)	0.81 (0.52, 1.28)	0.86 (0.53, 1.39)
Model 2	ref	1.30 (0.88, 1.94)	0.98 (0.66, 1.47)	1.08 (0.68, 1.70)
Model 3	ref	1.34 (0.88, 2.03)	1.08 (0.66, 1.75)	1.12 (0.67, 1.86)
Cancer-related mortality				
No. events (n)	85	65	59	56
Model 1	ref	0.56 (0.38, 0.83)	0.65 (0.41, 1.05)	0.43 (0.27, 0.69)
Model 2	ref	0.72 (0.46, 1.12)	0.85 (0.52, 1.39)	0.51 (0.32, 0.83)
Model 3	ref	0.71 (0.48, 1.06)	0.82 (0.49, 1.38)	0.51 (0.31, 0.82)
Flavanones (mg/day)	≤0.05	0.05–0.58	0.58–19.35	>19.35
Case/Person-years	1539/10,239	1526/10,994	1517/11,472	1528/11,273
All-cause mortality				
No. events (n)	293	272	265	325
Model 1	ref	0.81 (0.65, 1.02)	0.73 (0.57, 0.94)	0.81 (0.66, 1.00)
Model 2	ref	0.89 (0.70, 1.12)	0.89 (0.69, 1.14)	1.01 (0.82, 1.24)
Model 3	ref	0.92 (0.72, 1.16)	0.93 (0.73, 1.18)	1.04 (0.86, 1.27)
CVD-related mortality				
No. events (n)	91	58	63	70
Model 1	ref	0.62 (0.40, 0.97)	0.62 (0.37, 1.06)	0.58 (0.40, 0.85)
Model 2	ref	0.67 (0.41, 1.09)	0.75 (0.45, 1.26)	0.72 (0.49, 1.06)
Model 3	ref	0.67 (0.40, 1.13)	0.77 (0.48, 1.25)	0.76 (0.51, 1.14)
Cancer-related mortality				
No. events (n)	62	69	65	69
Model 1	ref	1.18 (0.76, 1.82)	0.80 (0.52, 1.25)	0.88 (0.54, 1.42)
Model 2	ref	1.31 (0.85, 2.01)	1.01 (0.64, 1.62)	1.20 (0.72, 2.00)
Model 3	ref	1.36 (0.88, 2.10)	1.06 (0.67, 1.69)	1.31 (0.75, 2.28)
Flavones(mg/day)	≤0.18	0.18–0.48	0.48–1.06	>1.06
Case/Person-years	1556/10,423	1499/10,907	1531/11,507	1524/11,140
All-cause mortality				
No. events (n)	329	303	295	228
Model 1	ref	0.84 (0.64, 1.10)	0.66 (0.51, 0.85)	0.54 (0.40, 0.74)
Model 2	ref	0.96 (0.74, 1.25)	0.80 (0.64, 1.01)	0.70 (0.51, 0.97)
Model 3	ref	0.96 (0.74, 1.26)	0.81 (0.65, 1.03)	0.72 (0.51, 1.03)
CVD-related mortality				
No. events (n)	77	79	72	54
Model 1	ref	0.93 (0.56, 1.53)	0.66 (0.39, 1.11)	0.46 (0.24, 0.90)
Model 2	ref	1.07 (0.64, 1.80)	0.82 (0.48, 1.40)	0.60 (0.30, 1.20)
Model 3	ref	1.09 (0.62, 1.92)	0.83 (0.49, 1.42)	0.61 (0.29, 1.29)
Cancer-related mortality				
No. events (n)	71	62	70	62
Model 1	ref	0.66 (0.38, 1.14)	0.65 (0.40, 1.04)	0.62 (0.34, 1.14)
Model 2	ref	0.73 (0.41, 1.29)	0.77 (0.46, 1.29)	0.79 (0.41, 1.53)
Model 3	ref	0.74 (0.42, 1.30)	0.82 (0.49, 1.38)	0.91 (0.45, 1.83)
Flavonols (mg/day)	≤6.58	6.58–12.17	12.17–21.55	>21.55
Case/Person-years	1528/10,420	1528/11,087	1526/10,964	1528/11,506
All-cause mortality				
No. events (n)	336	312	290	217
Model 1	ref	0.74 (0.60, 0.90)	0.73 (0.59, 0.89)	0.53 (0.39, 0.71)
Model 2	ref	0.80 (0.64, 1.00)	0.86 (0.69, 1.06)	0.63 (0.46, 0.86)
Model 3	ref	0.82 (0.64, 1.05)	0.87 (0.68, 1.12)	0.66 (0.46, 0.94)
CVD-related mortality				
No. events (n)	70	82	78	52
Model 1	ref	0.81 (0.49, 1.34)	0.78 (0.49, 1.24)	0.57 (0.29, 1.14)
Model 2	ref	0.88 (0.50, 1.53)	0.90 (0.52, 1.55)	0.72 (0.32, 1.58)
Model 3	ref	0.91 (0.51, 1.64)	0.94 (0.51, 1.73)	0.76 (0.33, 1.77)
Cancer-related mortality				
No. events (n)	78	74	61	52
Model 1	ref	0.81 (0.54, 1.20)	0.73 (0.44, 1.20)	0.46 (0.28, 0.77)
Model 2	ref	0.92 (0.63, 1.35)	0.86 (0.52, 1.42)	0.49 (0.28, 0.87)
Model 3	ref	0.93 (0.61, 1.42)	0.89 (0.52, 1.52)	0.52 (0.28, 0.96)

Hazard ratios (95% CI) for all-cause, CVD-related, and cancer-related mortality were analyzed using Cox proportional hazard models. Model 1: age (continuous, years) and sex (female and male). Model 2: model 1 + race (White, Black, Hispanic, Mexican American, and others), marital status (without partner and with partner), education status (<9 years, 9–12 years, and >12 years), activity (vigorous, moderate, and inactive), smoking status (never, former, and current), alcohol intake (continuous, g/d), BMI (continuous, kg/m^2^), and daily energy intake (continuous, kcal/d). Model 3: model 2 + total fruit intake (continuous, cup/d), total vegetable intake (continuous, cup/d), whole grains intake (continuous, oz/d), and red meat intake (continuous, oz/d).

**Table 4:** Hazard ratios of mortality by isoflavone intake groups.

Total Isoflavones (mg/day)	≤0.01	>0.01
Case/Person-years	3291/23,505	2819/20,473
All-cause mortality		
No. events (n)	699	456
HR (95% Cl)		
Model 1	ref	0.72 (0.61, 0.84)
Model 2	ref	0.77 (0.65, 0.92)
Model 3	ref	0.79 (0.67, 0.93)
CVD-related mortality		
No. events (n)	171	111
HR (95% Cl)		
Model 1	ref	0.67 (0.48, 0.94)
Model 2	ref	0.72 (0.51, 1.01)
Model 3	ref	0.73 (0.52, 1.03)
Cancer-related mortality		
No. events (n)	164	101
HR (95% Cl)		
Model 1	ref	0.75 (0.56, 0.99)
Model 2	ref	0.81 (0.59, 1.11)
Model 3	ref	0.82 (0.59, 1.13)

Hazard ratios (95% CI) for all-cause, CVD-related, and cancer-related mortality were analyzed using Cox proportional hazard models. Model 1: age (continuous, years) and sex (female and male). Model 2: model 1 + race (White, Black, Hispanic, Mexican American, and others), marital status (without partner and with partner), education status (<9 years, 9–12 years, and >12 years), activity (vigorous, moderate, and inactive), smoking status (never, former, and current), alcohol intake (continuous, g/d), BMI (continuous, kg/m^2^), and daily energy intake (continuous, kcal/d). Model 3: model 2 + total fruit intake (continuous, cup/d), total vegetable intake (continuous, cup/d), whole grains intake (continuous, oz/d), and red meat intake (continuous, oz/d).

## Data Availability

For detailed information regarding input data sources and to download the data used in these analyses, please visit the website at https://cdc.gov/nchs/nhanes/index.htm (accessed on 2 March 2024).

## Supplementary Materials

The following Supporting Information can be downloaded at: https://www.mdpi.com/article/10.3390/nu16101534/s1, Supplementary Table S1. Statistical description of flavonoid intakes. Supplementary Table S2. Pearson’s correlations between flavonoid subclasses. Supplementary Table S3. HRs and CIs of all-cause mortality, according to intake of 29 flavonoids. Supplementary Table S4. Hazard ratios of cancer-related mortality, according to intake of 29 flavonoids. Supplementary Table S5. Hazard ratios of CVD-related mortality, according to intake of 29 flavonoids. Supplementary Figure S1. HRs and 95% CIs for consumption of total flavonoids and cancer-related mortality, stratified by several key risk factors. Supplementary Figure S2. HRs and 95% CIs for consumption of total flavonoids and CVD-related mortality, stratified by several key risk factors. Supplementary Table S6. HRs and 95%CIs for risk of CVD according to intake of total flavonoid, adjusted for baseline history of chronic diseases. Supplementary Table S7. Hazard ratios for all-cause mortality by quartiles of total flavonoid intake after excluding participants with extreme energy intake (<500 and >4000 kcal/d). Supplementary Table S8. Hazard ratios for all-cause mortality by quartiles of total flavonoid intake after exclusion of participants with ≤1 year of follow-up.
