# Supplementary material for: Associations of Flavonoid Intakes with Mortality among Populations with Hypertension: A Prospective Cohort Study
Source: Nutrients. 2024 May 20;16(10):1534. doi: 10.3390/nu16101534 (PMC11124474; doi:10.3390/nu16101534)
Supplement: Supplementary file 1 [file nutrients-16-01534-s001.zip › nutrients-2978338-supplementary.pdf]

Online Supplementary Materials
Associations of flavonoid intakes with mortality among populations with hypertension: a prospective cohort study

Kang Wang, Taotao Lu, Rukai Yang, Shenghua Zhou

Supplementary Table S1. Statistical description of flavonoid intakes.
Supplementary Table S2. Pearson's correlations between flavonoid subclasses.
Supplementary Table S3. HRs and CIs of all-cause mortality, according to intake of 29 flavonoids.
Supplementary Table S4. Hazard ratios of cancer-related mortality, according to intake of 29 flavonoids.
Supplementary Table S5. Hazard ratios of CVD-related mortality, according to intake of 29 flavonoids.
Supplementary Figure S1. HRs and 95% CIs for consumption of total flavonoids and cancer-related mortality, stratified by several key risk factors.
Supplementary Figure S2. HRs and 95% CIs for consumption of total flavonoids and CVD-related mortality, stratified by several key risk factors.
Supplementary Table S6. HRs and 95%CIs for risk of CVD according to intake of total flavonoid, adjusted for baseline history of chronic diseases.
Supplementary Table S7. Hazard ratios for all-cause mortality by quartiles of total flavonoid intake after excluding participants with extreme energy intake (< 500 and > 4000 kcal/d).
Supplementary Table S8. Hazard ratios for all-cause mortality by quartiles of total flavonoid intake after exclusion of participants with ≤1 year of follow-up.

Supplementary Table S1. Statistical description of flavonoid intakes.

Intake of Flavonoids(mg/day)	Minimum	25th Percentile	Media	Mean	75th Percentile	Maximum
Total flavonoids	0	23.88	63.13	197.58	212.5	6974.47
Isoflavones	0	0	0.01	1.543	0.055	390.6
Anthocyanidins	0	0.1163	1.985	12.095	10.4037	640.89
Flavan-3-ols	0	4.902	15.18	153.078	153.292	6724.88
Flavanones	0	0.045	0.58	13.33	19.35	590.625
Flavones	0	0.175	0.4825	0.8045	1.055	24.315
Flavonols	0	6.576	12.165	16.732	21.551	239.93

Supplementary Table S2. Pearson's correlations between flavonoid subclasses.

	Total flavonoids	Flavonols	Flavanones	Anthocyanidins	Flavones	Isoflavones
Total flavonoids	1.00					
Flavonols	0.85*	1.00				
Flavanones	0.07*	0.06*	1.00			
Anthocyanidins	0.12*	0.18*	0.05*	1.00		
Flavones	0.28*	0.41*	0.11*	0.21*	1.00	
Isoflavones	0.02	-0.01	-0.01	0.02	0.01	1.00
Flavan-3-ols	0.99*	0.83*	-0.01	0.03*	0.24*	-0.01

*p<0.05

Supplementary Table S3. HRs and CIs of all-cause mortality, according to intake of 29 flavonoids.

Flavonoid species(mg/d)	Intake of flavonoids			
	Q1	Q2	Q3	Q4
Cyanidin	≤0.05	0.05-0.53	0.53-1.91	> 1.91
	1(reference)	0.83 (0.65, 1.06)	0.91 (0.73, 1.13)	0.81 (0.63, 1.05)
Peonidin	0	0-0.10	0.10-0.66	> 0.66
	1(reference)	0.97 (0.76, 1.25)	0.83 (0.66, 1.06)	0.76 (0.62, 0.92)
Catechin	≤1.76	1.76-4.97	4.97-9.71	> 9.71
	1(reference)	0.82 (0.65, 1.02)	0.79 (0.66, 0.94)	0.88 (0.70, 1.10)
Epigallocatechin	≤0.13	0.13-0.5	0.5-13.83	> 13.83
	1(reference)	0.84 (0.65, 1.08)	0.74 (0.60, 0.90)	0.69 (0.54, 0.89)
Epicatechin	≤1.36	1.36-5.52	5.52-12.22	> 12.22
	1(reference)	0.83 (0.62, 1.12)	0.87 (0.70, 1.09)	0.81 (0.63, 1.04)
Naringenin	≤0.03	0.03-0.30	0.30-3.51	> 3.51
	1(reference)	0.85 (0.67, 1.08)	1.00 (0.78, 1.29)	1.08 (0.84, 1.38)
Apigenin	≤0.02	0.02-0.07	0.07-0.22	> 0.22
	1(reference)	0.96 (0.75, 1.23)	0.85 (0.69, 1.04)	0.79 (0.66, 0.95)
Luteolin	≤0.11	0.11-0.33	0.33-0.8	> 0.8
	1(reference)	0.88 (0.70, 1.10)	0.75 (0.57, 0.98)	0.81 (0.59, 1.11)
Isorhamnetin	≤0.12	0.12-0.44	0.44-0.99	> 0.99
	1(reference)	0.94 (0.74, 1.19)	0.81 (0.63, 1.05)	0.84 (0.68, 1.05)
Kaempferol	≤0.83	0.83-2.24	2.24-5.32	> 5.32
	1(reference)	0.84 (0.66, 1.06)	0.77 (0.63, 0.95)	0.72 (0.54, 0.96)
Myricetin	≤0.26	0.26-0.6	0.6-1.63	> 1.63
	1(reference)	0.78 (0.61, 0.99)	0.70 (0.55, 0.88)	0.64 (0.48, 0.85)
Quercetin	≤4.21	4.21-7.85	7.85-13.52	> 13.52
	1(reference)	0.86 (0.66, 1.13)	0.93 (0.74, 1.18)	0.69 (0.52, 0.93)
Catechin	≤4.77	4.77-14.15	14.15-64.07	> 64.07
	1(reference)	0.92 (0.76, 1.11)	0.89 (0.73, 1.08)	0.80 (0.64, 1.00)
Genistein	≤0.005	> 0.005		
	1(reference)	0.82 (0.70, 0.96)		
Glycitein	0	> 0		
	1(reference)	0.69 (0.56, 0.86)		
Petunidin	0	> 0		
	1(reference)	0.87 (0.72, 1.05)		
Delphinidin	≤0.01	> 0.01		
	1(reference)	0.88 (0.73, 1.07)		
Malvidin	0	> 0		
	1(reference)	0.84 (0.70, 1.00)		
Pelargonidin	≤0.01	> 0.01		
	1(reference)	0.88 (0.75, 1.04)		
Epicatechin-3-gallate	≤0.025	> 0.025		
	1(reference)	0.82 (0.70, 0.97)		
Epigallocatechin-3-gallate	≤0.13	> 0.13		

	1(reference)	0.83 (0.69, 0.98)
Theaflavin	0	> 0
	1(reference)	0.82 (0.67, 1.00)
Thearubigins	0	> 0
	1(reference)	0.82 (0.67, 1.00)
Eriodictyol	0	> 0
	1(reference)	1.04 (0.87, 1.24)
Hesperetin	≤0.11	> 0.11
	1(reference)	1.01 (0.86, 1.18)
Theaflavin-3-3'-digallate	0	> 0
	1(reference)	0.83 (0.68, 1.01)
Theaflavin-3'-gallate	0	> 0
	1(reference)	0.83 (0.68, 1.01)
Theaflavin-3-gallate	0	> 0
	1(reference)	0.85 (0.69, 1.03)
Gallocatechin	≤0.015	> 0.015
	1(reference)	0.83 (0.71, 0.98)

Model adjusted for age(continuous, years), sex(female and male), race(White, Black, Hispanic, Mexican American and others), marital status(without partner and with partner), education status(<9 years, 9-12 years and >12 years), activity(vigorous, moderate and inactive), smoking status(never, former and current), alcohol intake(continuous, g/d), BMI(continuous, kg/m^2), daily energy intake(continuous, kcal/d), total fruit intake(continuous, cup/d), total vegetable intake(continuous, cup/d), whole grains intake(continuous, oz/d) and red meat intake(continuous, oz/d).

Supplementary Table S4. Hazard ratios of cancer-related mortality, according to intake of 29 flavonoids.

**Flavonoid species(mg/d)**	**Intake of flavonoids**			
	**Q1**	**Q2**	**Q3**	**Q4**
Cyanidin	≤0.05	0.05-0.53	0.53-1.91	> 1.91
	1	0.91 (0.50, 1.66)	0.88 (0.51, 1.53)	0.88 (0.48, 1.63)
Peonidin	0	0-0.10	0.10-0.66	> 0.66
	1	1.09 (0.63, 1.87)	0.78 (0.53, 1.15)	0.54 (0.36, 0.83)
Catechin	≤1.76	1.76-4.97	4.97-9.71	> 9.71
	1	0.47 (0.28, 0.76)	0.64 (0.42, 0.98)	0.72 (0.44, 1.20)
Epigallocatechin	≤0.13	0.13-0.5	0.5-13.83	> 13.83
	1	0.77 (0.48, 1.24)	0.84 (0.49, 1.42)	0.53 (0.33, 0.86)
Epicatechin	≤1.36	1.36-5.52	5.52-12.22	> 12.22
	1	0.69 (0.38, 1.27)	0.64 (0.35, 1.16)	0.62 (0.38, 1.01)
Naringenin	≤0.03	0.03-0.30	0.30-3.51	> 3.51
		1.23 (0.78, 1.93)	1.26 (0.78, 2.05)	1.19 (0.70, 2.01)
Apigenin	≤0.02	0.02-0.07	0.07-0.22	> 0.22
		0.97 (0.61, 1.55)	0.83 (0.52, 1.32)	0.98 (0.63, 1.54)
Luteolin	≤0.11	0.11-0.33	0.33-0.80	> 0.80
		0.75 (0.46, 1.22)	0.63 (0.36, 1.12)	0.94 (0.48, 1.82)
Isorhamnetin	≤0.12	0.12-0.44	0.44-0.99	> 0.99
		0.73 (0.42, 1.26)	0.56 (0.33, 0.96)	0.87 (0.60, 1.26)
Kaempferol	≤0.83	0.83-2.24	2.24-5.32	> 5.32
		0.88 (0.53, 1.48)	0.55 (0.33, 0.91)	0.51 (0.32, 0.82)
Myricetin	≤0.26	0.26-0.6	0.6-1.63	> 1.63
	1	0.80 (0.52, 1.24)	0.66 (0.39, 1.10)	0.44 (0.26, 0.76)
Quercetin	≤4.21	4.21-7.85	7.85-13.52	> 13.52
	1	0.89 (0.57, 1.39)	0.94 (0.59, 1.50)	0.55 (0.34, 0.88)
Catechin	≤4.77	4.77-14.15	14.15-64.07	> 64.07
	1	0.64 (0.39, 1.03)	0.81 (0.49, 1.33)	0.52 (0.33, 0.81)
Genistein	≤0.005	> 0.005		
	1	0.87 (0.63, 1.19)		
Glycitein	0	> 0		
	1	0.94 (0.62, 1.44)		
Petunidin	0	> 0		
	1	0.73 (0.52, 1.02)		
Delphinidin	≤0.01	> 0.01		
	1	0.61 (0.45, 0.84)		
Malvidin	0	> 0		
	1	0.67 (0.47, 0.94)		
Pelargonidin	≤0.01	> 0.01		
	1	0.93 (0.67, 1.29)		
Epicatechin-3-gallate	≤0.025	> 0.025		
	1	0.73 (0.52, 1.01)		
Epigallocatechin-3-gallate	≤0.13	> 0.13		

	1	0.73 (0.53, 1.01)
Theaflavin	0	> 0
	1	0.64 (0.45, 0.91)
Thearubigins	0	> 0
	1	0.64 (0.45, 0.92)
Eriodictyol	0	> 0
		1.32 (0.93, 1.87)
Hesperetin	≤0.11	> 0.11
		1.01 (0.74, 1.38)
Theaflavin-3-3'-digallate	0	> 0
	1	0.65 (0.45, 0.92)
Theaflavin-3'-gallate	0	> 0
	1	0.65 (0.46, 0.92)
Theaflavin-3-gallate	0	> 0
	1	0.67 (0.47, 0.95)
Gallocatechin	≤0.015	> 0.015
	1	0.66 (0.47, 0.92)

Model adjusted for age(continuous, years), sex(female and male), race(White, Black, Hispanic, Mexican American and others), marital status(without partner and with partner), education status(<9 years, 9-12 years and >12 years), activity(vigorous, moderate and inactive), smoking status(never, former and current), alcohol intake(continuous, g/d), BMI(continuous, kg/m^2), daily energy intake(continuous, kcal/d), total fruit intake(continuous, cup/d), total vegetable intake(continuous, cup/d), whole grains intake(continuous, oz/d) and red meat intake(continuous, oz/d).

Supplementary Table S5. Hazard ratios of CVD-related mortality, according to intake of 29 flavonoids.

Flavonoid species(mg/d)	Intake of flavonoids			
	Q1	Q2	Q3	Q4
Cyanidin	≤0.05	0.05-0.53	0.53-1.91	> 1.91
	1	0.92 (0.49, 1.71)	0.93 (0.54, 1.62)	0.58 (0.29, 1.16)
Peonidin	0	0-0.10	0.10-0.66	> 0.66
	1	0.95 (0.51, 1.78)	0.88 (0.54, 1.44)	0.72 (0.39, 1.33)
Catechin	≤1.76	1.76-4.97	4.97-9.71	> 9.71
	1	1.19 (0.77, 1.85)	0.99 (0.70, 1.39)	1.18 (0.73, 1.92)
Epigallocatechin	≤0.13	0.13-0.5	0.5-13.83	> 13.83
	1	0.85 (0.44, 1.64)	0.72 (0.41, 1.26)	0.78 (0.40, 1.53)
Epicatechin	≤1.36	1.36-5.52	5.52-12.22	> 12.22
	1	1.09 (0.65, 1.82)	1.11 (0.70, 1.74)	0.93 (0.54, 1.60)
Naringenin	≤0.03	0.03-0.30	0.30-3.51	> 3.51
		0.69 (0.49, 0.97)	0.85 (0.49, 1.50)	0.87 (0.54, 1.38)
Apigenin	≤0.02	0.02-0.07	0.07-0.22	> 0.22
		1.48 (0.93, 2.36)	0.96 (0.62, 1.47)	0.96 (0.58, 1.58)
Luteolin	≤0.11	0.11-0.33	0.33-0.80	> 0.80
		0.82 (0.52, 1.29)	0.77 (0.39, 1.54)	0.66 (0.35, 1.27)
Isorhamnetin	≤0.12	0.12-0.44	0.44-0.99	> 0.99
		0.88 (0.54, 1.43)	1.11 (0.69, 1.79)	0.86 (0.53, 1.38)
Kaempferol	≤0.83	0.83-2.24	2.24-5.32	> 5.32
		0.78 (0.48, 1.26)	0.93 (0.62, 1.40)	0.87 (0.48, 1.59)
Myricetin	≤0.26	0.26-0.60	0.60-1.63	> 1.63
	1	0.81 (0.47, 1.41)	0.77 (0.52, 1.15)	0.70 (0.38, 1.31)
Quercetin	≤4.21	4.21-7.85	7.85-13.52	> 13.52
	1	0.76 (0.42, 1.37)	0.96 (0.55, 1.65)	0.74 (0.34, 1.59)
Catechin	≤4.77	4.77-14.15	14.15-64.07	> 64.07
	1	1.24 (0.82, 1.88)	1.12 (0.71, 1.77)	1.13 (0.71, 1.81)
Genistein	≤0.005	> 0.005		
	1	0.76 (0.55, 1.07)		
Glycitein	0	> 0		
	1	0.59 (0.38, 0.93)		
Petunidin	0	> 0		
	1	0.88 (0.58, 1.31)		
Delphinidin	≤0.01	> 0.01		
	1	0.84 (0.57, 1.25)		
Malvidin	0	> 0		
	1	0.79 (0.57, 1.11)		
Pelargonidin	≤0.01	> 0.01		
	1	0.79 (0.55, 1.13)		
Epicatechin-3-gallate	≤0.025	> 0.025		
	1	0.85 (0.59, 1.23)		
Epigallocatechin-3-gallate	≤0.13	> 0.13		

	1	0.92 (0.65, 1.30)
Theaflavin	0	> 0
	1	0.96 (0.61, 1.51)
Thearubigins	0	> 0
		0.96 (0.61, 1.52)
Eriodictyol	0	> 0
		0.89 (0.60, 1.33)
Hesperetin	≤0.11	> 0.11
		0.78 (0.52, 1.17)
Theaflavin-3-3'-digallate	0	> 0
	1	0.97 (0.62, 1.54)
Theaflavin-3'-gallate	0	> 0
	1	0.98 (0.62, 1.54)
Theaflavin-3-gallate	0	> 0
	1	1.00 (0.63, 1.60)
Gallocatechin	≤0.015	> 0.015
	1	0.87 (0.60, 1.26)

Model adjusted for age(continuous, years), sex(female and male), race(White, Black, Hispanic, Mexican American and others), marital status(without partner and with partner), education status(<9 years, 9-12 years and >12 years), activity(vigorous, moderate and inactive), smoking status(never, former and current), alcohol intake(continuous, g/d), BMI(continuous, kg/m^2), daily energy intake(continuous, kcal/d), total fruit intake(continuous, cup/d), total vegetable intake(continuous, cup/d), whole grains intake(continuous, oz/d) and red meat intake(continuous, oz/d).

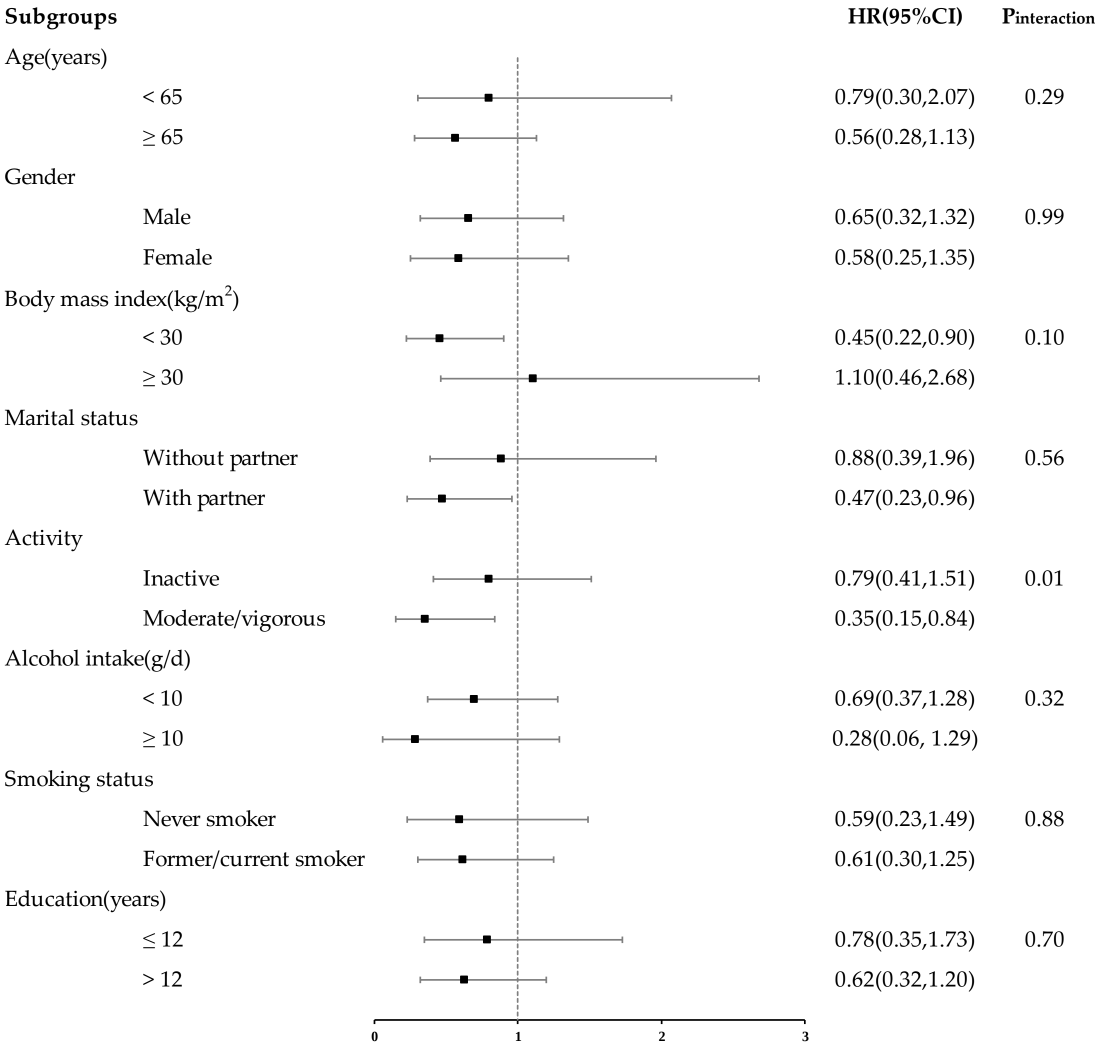

Supplementary Figure S1. HRs and 95% CIs for consumption of total flavonoids and cancer-related mortality, stratified by several key risk factors.

All models were multivariable adjusted for age, sex, race, marital status, education status, activity, smoking status, alcohol intake, BMI, daily energy intake, total fruit intake, total vegetable intake, whole grains intake and red meat intake. In each stratified analysis, the stratification variable was excluded in the adjustments. The HR and 95%CI of each subgroup in the figure from the group with the highest intake of total flavonoids. Likelihood ratio tests were used for assessment of interaction, and two-sided P values (unadjusted for multiple comparisons) are reported.

Subgroups	HR(95%CI)	$P_{interaction}$
Age(years)		
< 65	0.81(0.29,2.27)	0.68
≥ 65	0.83(0.44,1.57)	
Gender		
Male	0.77(0.32,1.86)	0.91
Female	0.71(0.27,1.84)	
Body mass index(kg/m^2)		
< 30	0.43(0.19,0.99)	0.08
≥ 30	1.34(0.58,3.10)	
Marital status		
Without partner	0.84(0.45,1.56)	0.25
With partner	0.70(0.30,1.66)	
Activity		
Inactive	0.92(0.50,1.71)	0.37
Moderate/vigorous	0.57(0.14,2.34)	
Alcohol intake(g/d)		
< 10	0.86(0.42,1.74)	0.19
≥ 10	0.33(0.09,1.27)	
Smoking status		
Never smoker	1.20(0.51,2.79)	0.29
Former/current smoker	0.50(0.25,1.00)	
Education(years)		
≤ 12	0.84(0.32,2.25)	0.64
> 12	0.69(0.35,1.37)	

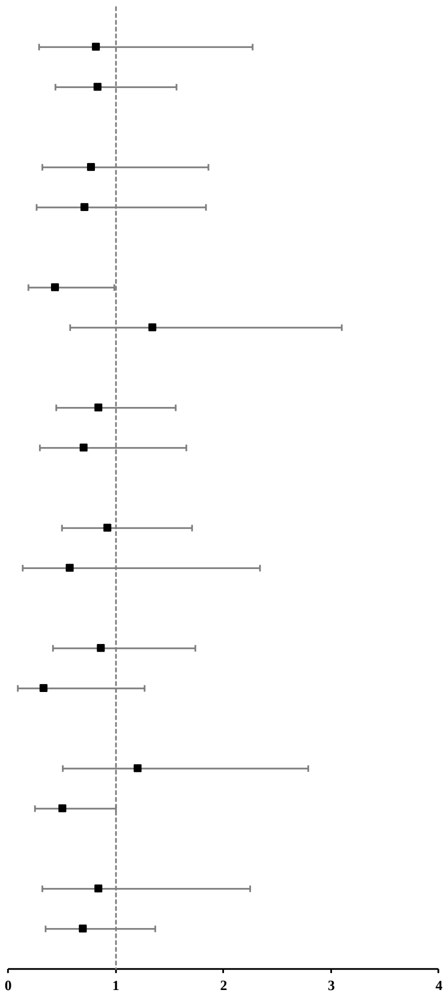

Supplementary Figure S2. HRs and 95% CIs for consumption of total flavonoids and CVD-related mortality, stratified by several key risk factors.

All models were multivariable adjusted for age, sex, race, marital status, education status, activity, smoking status, alcohol intake, BMI, daily energy intake, total fruit intake, total vegetable intake, whole grains intake and red meat intake. In each stratified analysis, the stratification variable was excluded in the adjustments. The HR and 95%CI of each subgroup in the figure from the group with the highest intake of total flavonoids. Likelihood ratio tests were used for assessment of interaction, and two-sided P values (unadjusted for multiple comparisons) are reported.

Supplementary Table S6. HRs and 95%CIs for risk of CVD according to intake of total flavonoid, adjusted for baseline history of chronic diseases.

Frequencies of intake	Event/person-years	HR (95%CI)
Q1	318/10,707	1 (reference)
Q2	286/11,071	0.85 (0.69, 1.05)
Q3	294/10,706	0.74 (0.56, 0.98)
Q4	257/11,494	0.77 (0.58, 0.99)

Based on the adjustment for age, sex, race, marital status, education status, activity, smoking status, alcohol intake, BMI, daily energy intake, total fruit intake, total vegetable intake, whole grains intake and red meat intake. This multivariable model was additionally adjusted for history of diabetes and hyperlipidemia (yes or no).

Supplementary Table S7. Hazard ratios for all-cause mortality by quartiles of total flavonoid intake after excluding participants with extreme energy intake (< 500 and > 4000 kcal/d).

	Total flavonoid intake			
	Q1	Q2	Q3	Q4
Model 1	1 (reference)	0.76 (0.64, 0.91)	0.59 (0.48, 0.72)	0.61 (0.48, 0.77)
Model 2	1 (reference)	0.88 (0.72, 1.07)	0.75 (0.59, 0.97)	0.78 (0.61, 0.99)
Model 3	1 (reference)	0.87 (0.70, 1.07)	0.72 (0.54, 0.96)	0.76 (0.58, 0.99)

Model 1: age and sex. Model 2: model 1 + race, marital status, education status, activity, smoking status, alcohol intake, BMI and daily energy intake. Model 3: model 2 + total fruit intake, total vegetable intake, whole grains intake and red meat intake.

Supplementary Table S8. Hazard ratios for all-cause mortality by quartiles of total flavonoid intake after exclusion of participants with ≤1 year of follow-up.

	Total flavonoid intake			
	Q1	Q2	Q3	Q4
Model 1	1 (reference)	0.78 (0.64, 0.94)	0.61 (0.50, 0.75)	0.59 (0.47, 0.74)
Model 2	1 (reference)	0.88 (0.71, 1.10)	0.76 (0.60, 0.98)	0.73 (0.57, 0.95)
Model 3	1 (reference)	0.88 (0.69, 1.11)	0.73 (0.55, 0.97)	0.72 (0.54, 0.95)

Model 1: age and sex. Model 2: model 1 + race, marital status, education status, activity, smoking status, alcohol intake, BMI and daily energy intake. Model 3: model 2 + total fruit intake, total vegetable intake, whole grains intake and red meat intake.
